# Analysis of COVID‐19 vaccines: Types, thoughts, and application

**DOI:** 10.1002/jcla.23937

**Published:** 2021-08-15

**Authors:** Xiucui Han, Pengfei Xu, Qing Ye

**Affiliations:** ^1^ Department of Clinical Laboratory The Children’s Hospital Zhejiang University School of Medicine National Clinical Research Center for Child Health Hangzhou China; ^2^ Clinical Laboratory Zhejiang Hospital Hangzhou China

**Keywords:** application, COVID‐19, thoughts, types, vaccine

## Abstract

**Objective:**

To deal with COVID‐19, various countries have made many efforts, including the research and development of vaccines. The purpose of this manuscript was to summarize the development, application, and problems of COVID‐19 vaccines.

**Methods:**

This article reviewed the existing literature to see the development of the COVID‐19 vaccine.

**Results:**

We found that different types of vaccines had their own advantages and disadvantages. At the same time, the side effects of the vaccine, the dose of vaccination, the evaluation of the efficacy, and the application of the vaccine were all things worth studying.

**Conclusion:**

The successful development of the COVID‐19 vaccine concerns almost all countries and people in the world. We must do an excellent job of researching the immunogenicity and immune reactivity of the vaccines. We hope this review can help colleagues at home and abroad.

## Graphical abstract

We reviewed the mechanism and types of COVID‐19 vaccines through the literature. At the same time, we found some existing problems in vaccines research, such as injection dosage, adverse reactions, efficacy evaluation and vaccine application. An excellent job must be performed to research the immunogenicity and immune reactivity of the COVID‐19 vaccines.

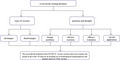

## 1 INTRODUCTION

On December 31, 2019, a novel coronavirus disease caused by severe acute respiratory syndrome type 2 coronavirus (SARS‐CoV‐2) was first reported in China.^1^ On January 30, 2020, the World Health Organization (WHO) announced that the new coronavirus pneumonia epidemic was listed as a "public health emergency of international concern," and on February 11, the WHO officially named the disease as Coronavirus disease 2019 (COVID‐19).^2^ COVID‐19 had a devastating impact on almost all countries in the world. Because the new coronavirus is highly contagious and spreads quickly, it is not easy to find in mild cases and asymptomatic infections. Also, it is easy to cause "hidden" transmission in communities and medical institutions. The virus may gradually evolve into a seasonal low‐level epidemic. Even if the virus can be completely eliminated from the population, the transmission mechanism from the host to the person is still unclear due to the population's general susceptibility. There is a risk of recurrence or periodic epidemics. Vaccines need to be administered as soon as possible. Globally, 7.8 billion people are at risk of SARS‐CoV‐2 infection and the morbidity and death of COVID‐19. People are looking forward to developing an effective and safe COVID‐19 vaccine to contain this COVID‐19 pandemic and prevent another outbreak of the epidemic. More than 200 COVID‐19 vaccines have been listed in the WHO as under development. The expectations for effective prophylactic COVID‐19 vaccines are very high.^3^ Vaccines proven effective and safe in phase III clinical trials may enter the market in 2021.^4^ Several vaccines have been approved for marketing, such as Pfizer‐BioNTech's BNT162b2 and Moderna's mRNA‐1273.

## 2 MAIN MECHANISM

The design of the COVID‐19 vaccines must take into account both humoral and cellular immunity. In addition, COVID‐19 is mainly spread through the respiratory tract and contact, so the role of mucosal immunity in preventing viral infections should be paid more attention. The virus contains four structural proteins. They are Spike S protein, Envelope E protein, Membrane/matrix protein, and Nucleocapsid N protein. The S protein has two subsections, S1 and S2. The S protein binds to specific receptors, causing the virus to infect cells.^5^, ^6^, ^7^ The neutralizing antibody against the S protein can block this process and prevent the virus from invading.^8^ S protein can also effectively stimulate T‐cell immune response, so it is the most important target antigen for vaccine design. N and M proteins have also been shown to induce the body to produce an efficient cellular immune response.^9^, ^10^, ^11^

SARS‐CoV‐2 is unusual for a respiratory virus that binds to a receptor, angiotensin‐converting enzyme 2 (ACE2). ACE2 can be expressed in virtually all organs, but especially in the lungs,^12^ gut,^13^ and brain.^14^ Therefore, unlike most respiratory viruses, SARS‐CoV‐2 has a wider biological distribution and may cause considerable damage outside the respiratory system. It adversely affects the genitourinary system, digestive system, circulatory system, and central nervous system. The universality of the distribution of ACE2 receptors leads to multiple changes in symptoms, such as dyspnea, headache, diarrhea, venous thromboembolism, and high blood pressure.^15^ The S protein binds to ACE2 on cells to mediate infection. The S1 subunit contains the receptor‐binding domain (RBD) and is responsible for initial attachment to the host cells through the ACE2 receptor, while the S2 subunit promotes viral fusion with cells to initiate infection.^16^ The S protein is a frequent vaccine target as it is expected that antibodies binding to the correct epitope on the S protein may be neutralizing and block intercellular viral spread.^16^

## 3 TYPES OF VACCINES

The vaccines currently under study can be roughly divided into the following categories. Different types of vaccines have their characteristics (Table 1).

**TABLE 1:** Introduction to several types of vaccines

Types of vaccines	Mechanisms	Advantages	Disadvantages	Examples
DNA vaccines	DNA vaccines can enter cells like viral infections and use the host protein translation system to generate target antigens. It can induce humoral and cellular immune responses at the same time.	DNA vaccines do not require live viruses. The manufacturing process of plasmid DNA is relatively straightforward, and the double‐strand DNA molecules are more stable than the virus and can be freeze‐dried for long‐term storage.	DNA vaccine vaccination method limits its application	INO−4800
mRNA vaccines	mRNA vaccines need to enter the cytoplasm to achieve target antigens’ expression	theoretically safer	The immune effect is not satisfactory, and a relatively high proportion of side effects.	mRNA−1273
Non‐replicating viral vector vaccines	It can encode for the full‐length S protein of SARS‐CoV−2.	The effectiveness of this vaccine is relatively high.	It may not effective for people with recessive infectious viruses.	Ad5‐nCoV
Inactivated vaccines	Inactivated vaccines are mainly obtained through three inactivation methods, which make it lose its infectivity and toxicity while maintaining immunogenicity.	They are easy to prepare and can efficiently cause humoral immune responses.	Vaccine production requires the operation of high concentrations of live viruses, which poses a certain biological safety risk. The T‐cell immune response caused by inactivated vaccines is generally weak.	The inactivated SARS‐CoV−2 vaccine (Vero cells)
Live attenuated vaccines	Live attenuated vaccine reduces virus virulence through point mutation or deletion of crucial virus protein but does not affect its immunogenicity and replication ability.	This vaccine program has very good immunogenicity and can induce systemic immunity and mucosal immune response, and the immunity is lasting.	It is possible to restore virulence in the body due to retrograde mutations	Polio vaccine
Subunit vaccines	The antigen protein of the pathogen is expressed and purified through genetic engineering to induce an immune response.	Subunit vaccines are composed of purified recombinant proteins and are considered to be the safest vaccines. It has good safety and immunogenicity.	As a non‐endogenous antigen, subunit vaccines cannot be presented through MHC‐I and cannot effectively produce sensitized cytotoxic T cells (CTL).	Recombinant subunit SARS‐CoV−2 vaccine (CHO cells)
Trained immunity‐based vaccines	Trained immunity‐based vaccines can activate the adaptive immune system and provide pathogen‐specific protection	/	The production standards of the BCG vaccine will be different.	BCG vaccine

### 3.1 DNA vaccines

DNA vaccines can enter cells like viral infections and use the host protein translation system to generate target antigens. As an endogenous immunogen, it can induce humoral and cellular immune responses at the same time. Given the advantages of nucleic acid vaccines, DNA vaccines do not require live viruses, so safety is improved. DNA vaccines insert genes encoding foreign antigens into plasmids containing eukaryotic expression elements and then directly introduce the plasmids into humans or animals, allowing them to express antigen proteins in host cells and induce immune responses to prevent diseases.^17^

The manufacturing process of plasmid DNA is relatively straightforward, and the double‐strand DNA molecules are more stable than the virus and can be freeze‐dried for long‐term storage. DNA vaccine vaccination method limits its application. Since the vaccine is mainly distributed in the intercellular space after vaccination, only a very small amount can enter the cell to produce protein immunogen, so the immune effect is greatly reduced. The plasmid DNA vaccine's main prohibitory factor is the low transfection efficacy, which requires transfection modalities. For example, Inovio's COVID‐19 vaccine candidate, INO‐4800, uses a handheld electroporation device, CELLECTRA.^18^ The vaccine will be injected intradermally along with the electrodes. An electric pulse is then applied to open the cell membrane so that the plasmid can enter the cells. Using an established device may allow fast launch in clinical trials, but it also brings other obstacles to large‐scale vaccination. Although nucleic acid vaccines can effectively induce systemic immune responses, their immunogenicity is weak, and mucosal immune responses are not easy to produce. Although a few animal DNA vaccines have been on the market, no human DNA vaccine has been approved for marketing so far. Combination with other vaccines will achieve better immune effects.

### 3.2 mRNA vaccines

Compared with DNA vaccines that need to enter the nucleus, mRNA vaccines only need to enter the cytoplasm to achieve target antigens’ expression, so they are theoretically safer. In recent years, mRNA vaccines have been developed rapidly. Although the mRNA vaccines for rabies virus and influenza virus have completed phase I clinical evaluation,^19^, ^20^ the immune effect is not satisfactory, such as a relatively high proportion of headaches, fatigue, and side effects such as muscle pain. The immune protection generated by the vaccine declined rapidly within one year, and no cellular immune response was detected. Therefore, it is necessary to improve further the immune efficacy and long‐term protection of mRNA vaccines. So far, there is no mRNA vaccine on the market. However, the research of mRNA vaccines has been in the process of exploration and advancement. Many institutions at home and abroad have quickly initiated the research and development of COVID‐19 mRNA vaccines. The mRNA vaccine developed by the National Institute of Allergy and Infectious Diseases (NIAID) and Moderna has taken the lead to initiate a phase I clinical trial. Moderna's vaccine, mRNA‐1273, specifically encodes the S antigen's prefusion form, including a transmembrane anchor and an entire S1−S2 cleavage site.^21^

### 3.3 Non‐replicating viral vector vaccines

One of the most explored viral vector options is the Adenovirus (Ad), currently being used by both CanSino and Oxford/ AstraZeneca. Adenovirus is common cold viruses with a double‐stranded DNA genome. CanSino is using Ad type 5 (Ad5) and named the vaccine Ad5‐nCoV.^22^ Ad5‐nCoV can encode for the full‐length S protein of SARS‐CoV‐2. This gene is derived from the Wuhan‐Hu‐1 sequence of SARS‐CoV‐2 and is cloned into the E1‐ and E3‐deleted Ad5 vector together with the tissue plasminogen activator signal peptide.^16^ The effectiveness of this vaccine is relatively high, but the disadvantage is that it may not effective for people with recessive infectious viruses.

### 3.4 Inactivated vaccines

Inactivated vaccines are the most classic form of vaccines. They are easy to prepare and can efficiently cause humoral immune responses. They are often the first choice for new infectious diseases. Inactivated vaccines are mainly obtained through three inactivation methods, such as formaldehyde, β‐propiolactone, and ultraviolet. SARS and MERS inactivated vaccines can cause mice, hamsters, ferrets, and monkeys to produce high‐titer neutralizing antibodies. The SARS‐inactivated vaccine has completed phase I clinical trials, proving that it is safe in humans and can induce neutralizing antibodies’ production.^23^ However, the T‐cell immune response caused by inactivated vaccines is generally weak. Previous studies have shown that SARS‐ and MERS‐inactivated vaccines cannot effectively stimulate the body to produce cellular immune responses.^24^, ^25^ Although high titers of serum neutralizing antibodies are produced, the protective effect is also not satisfied.^25^ Some studies have found that the MERS‐inactivated vaccine can cause pathological allergic reactions in mice's lungs.^26^ Currently, the inactivated SARS‐CoV‐2 vaccine (Vero cells) is being used. In addition, vaccine production requires the operation of high concentrations of live viruses, which poses a certain biological safety risk.

### 3.5 Live attenuated vaccines

Live attenuated vaccine reduces virus virulence through point mutation or deletion of crucial virus protein but does not affect its immunogenicity and replication ability. This vaccine program has very good immunogenicity and can induce systemic immunity and mucosal immune response, and the immunity is lasting. Several live attenuated vaccines have been on the market, including yellow fever, smallpox, measles, polio, mumps, rubella, and chickenpox. The SARS live attenuated vaccine will recover its virulence after continuous passage in cells or mice, suggesting that the vaccine scheme has a greater biological safety risk.^27^ Without sufficient evidence to ensure that live attenuated vaccines will not regain strength, this strategy is not currently recommended for COVID‐19 vaccine development.

### 3.6 Subunit vaccines

Subunit vaccines are composed of purified recombinant proteins and are considered to be the safest vaccines. There are currently several subunit vaccines on the market, including hepatitis B, hepatitis E, and human papillomavirus vaccines. SARS and MERS subunit vaccines can produce high‐titer neutralizing antibodies in mice, and nasal or oral vaccination can also induce a mucosal immune response, thereby more effectively blocking the virus transmission through the respiratory tract. The data also prove the protective efficacy of mucosal vaccination better than intramuscular inoculation.^28^, ^29^, ^30^, ^31^ However, as a non‐endogenous antigen, subunit vaccines cannot be presented through MHC‐I and cannot effectively produce sensitized cytotoxic T cells (CTL). Considering the key role of cellular immunity in clearing coronavirus infections, the subunit vaccine of COVID‐19 is best used in conjunction with other platform vaccines. It is recommended to include nasal and oral mucosal vaccination routes to activate mucosal immune responses.

### 3.7 Trained immunity‐based vaccines

Trained immunity‐based vaccines can activate the adaptive immune system and provide pathogen‐specific protection.^32^, ^33^ Currently, Bacille Calmette‐Guerin (BCG), a vaccine against tuberculosis, can induce trained immunity against COVID‐19 and is currently undergoing clinical evaluation, which will take time to prove.^34^ Even if the BCG vaccine is effective against COVID‐19, it also faces unique challenges. That is, the production standards of the BCG vaccine will vary from country to country, and it is not clear whether certain quality standards are required to provide protection against COVID‐19.^35^

## 4 QUESTIONS AND THOUGHTS

There are always various problems in the development of vaccines. Among them, the later application and evaluation of vaccines are particularly prominent.

### 4.1 Dosage problems

Many phase III studies failed because of incorrect identification of the dose that best balances safety and efficacy.^36^ For example, the dosing regimen for the mRNA vaccine is still under study. The 250μg dose does not seem to have significantly higher antibody titers than the 100μg dose, but it is related to a higher proportion of serious systemic adverse events. As the researchers pointed out, carefully evaluate doses of 100 μg or lower to define the regimen that provides the most appropriate benefit‐risk profile for the vaccine. In this case, another special dosage consideration is age. The immune function that declines with age may lead to a greater risk of severe COVID‐19 in the elderly and lead to low vaccine responses. As observed in the influenza vaccine, is a large dose of the COVID‐19 vaccine needed to protect the elderly^37^ effectively? It may take some time to solve these problems.

### 4.2 Adverse reactions of vaccines

The vaccine also has adverse reactions such as redness, swelling, muscle pain, and fever.^38^, ^39^ The strategic objectives of the COVID‐19 vaccine roadmap formulated by WHO include a series of preferred and most basic requirements such as vaccine safety and effectiveness.^40^ The preferred requirements for safety/reactogenicity include "safety and reactogenicity sufficient to provide a highly favorable benefit/risk profile in the context of the observed vaccine efficacy and only mild, transient related to adverse vaccination events without serious adverse events". The most basic requirements for safety/reactogenicity include the benefits of vaccines outweigh the potential safety hazards. The long‐term results were a safety that is sufficient to provide highly favorable benefits/risk characteristics in the context of the observed vaccine efficacy and immunogenicity. There were no serious adverse events related to vaccination. The preferred requirements for effectiveness include the protection effectiveness in the population is at least 70%, and the same is true for the elderly. If it is an outbreak treatment, the protective effect must appear within two weeks and last for at least one year. The most basic requirements include the population's protective effect is at least about 50% for at least six months.

### 4.3 Clinical trials and Efficacy evaluation (endpoint observation)

Key considerations for the safety of COVID‐19 vaccine research and development were important. Most of the preventive vaccines’ targets are healthy individuals, and their safety is the most important issue in vaccine development and clinical trials. The State Drug Administration has issued the "Notice on the Guidelines for the Classification of Adverse Events in Clinical Trials of Preventive Vaccines"^41^ and other relevant regulations to guide vaccine development and clinical trials. In the past, in the development of inactivated measles vaccines and respiratory syncytial virus vaccines, there has been an increase in antibody‐dependent infection.^42^ Therefore, when studying COVID‐19 vaccines, attention should be paid to whether they will cause similar immunopathological reactions. Long‐term safety observations are required.

Although the Food and Drug Administration (FDA) of the United States recommends defining the COVID‐19 endpoint as virologically confirmed SARS‐CoV‐2 infection accompanied by one or more of 11 Symptoms,^43^ trialists have the latitude in selecting specific symptoms and severities to trigger virologic testing. It is important to define a common COVID‐19 endpoint that can be used consistently across trials, not only to interpret results but also to facilitate trials’ meta‐analyses.

It is very important to use a standard set of clinical endpoints for vaccine efficacy evaluation in all trials to conduct a unified and comprehensive assessment of benefits and risks support aggregated data to analyze the immunosurrogate endpoint. In any case, COVID‐19 and severe COVID‐19 should be important independent clinical endpoints to be evaluated in each vaccine efficacy trial. All participants should be followed up sufficiently to collect meaningful data to evaluate long‐term protection against these two endpoints, especially the severe COVID‐19. More endpoint counts are needed to quantify vaccine efficacy reliably. Considering that vaccine‐induced decrease in the incidence of symptomatic SARS‐CoV‐2 infections may be accompanied by a shift toward more asymptomatic infections, the ability to evaluate vaccine efficacy against the asymptomatic infection endpoint in trial designs should be included.

### 4.4 Vaccine application

Preventive vaccines will control COVID‐19 is justified by the impact of vaccines on preventing disability and death from infectious diseases.^44^, ^45^ Even if vaccine supply is no longer an obstacle, people's hesitation to vaccinate remains a major challenge.

In first‐line medical staff at the University of Texas Southwestern Medical Center, 234 of the 8,969 unvaccinated employees were infected (2.61%), and only 4 of the 8,121 vaccinated employees were infected (0.05%), and one research was in California also got similar results.^46^, ^47^

In a hospital in Jerusalem, among the medical staff who received two doses of the vaccine, the number of new COVID‐19 cases had decreased significantly despite the sudden increase of the B.1.1.7 variant (see up to 80% of cases). These data show that vaccination had largely protected frontline medical workers in high‐risk environments.^48^ Therefore, vaccination is particularly important in global epidemic prevention work.

### 4.5 Travel immunization

If the epidemic situation is well controlled, and the future epidemic situation is mainly imported, entry and exit personnel should be the target of implementing the immunization strategy, and close contacts of entry personnel should be used as vaccinations.

### 4.6 Immunization after exposure

If it is confirmed that the COVID‐19 vaccine has the effect of preventing or alleviating the symptoms of the disease on the exposed subjects, it is possible to consider adopting a post‐exposure immunization strategy for close contacts of confirmed COVID‐19 cases. Therefore, it is necessary to evaluate the protective effect of COVID‐19 vaccines, especially vaccines developed by new technologies, to determine post‐exposure vaccination's scientific nature.

### 4.7 Pre‐exposure immunization

For subjects who may be exposed to COVID‐19 patients or high‐risk infections, such as medical staff in fever clinics, COVID‐19 pathogen testing personnel, contact persons from COVID‐19 endemic countries, etc., should take exposure pre‐immune prevention strategies.

### 4.8 Emergency immunization

In the event of a COVID‐19 epidemic, under the premise of confirming the emergency immunization effect of the COVID‐19 vaccine, an emergency immunization strategy can be considered for the population in the epidemic area. Therefore, in the early stage of vaccine marketing, it is necessary to carry out the effect evaluation of emergency immunization, especially the effect evaluation of the ring immunization strategy's implementation.

In addition to the above four immunization strategies for pandemic immunization, because COVID‐19 is susceptible to the entire population, this feature is different from the H1N1 pandemic.^49^ In the case of vaccine supply in batches, comprehensive consideration of protection priorities, reducing deaths and cluster outbreaks determine the targets and immunization procedures for mass pandemic vaccination.

## 5 CONCLUSION

In short, the successful development of the COVID‐19 vaccine concerns almost all countries and people in the world. We must do an excellent job of researching the immunogenicity and immune reactivity of the vaccines.

## CONFLICTS OF INTEREST

The authors declare no conflicts of interest.

## AUTHORS CONTRIBUTIONS

XCH designed the current study and was major contributors in writing the manuscript. PFX and QY were responsible for the modification and giving final approval of the manuscript. All authors read and approved the final manuscript.

## Data Availability

Data sharing is not applicable to this article as no new data were created or analyzed in this study.
